# Fish Oil, Cannabidiol and the Gut Microbiota: An Investigation in a Murine Model of Colitis

**DOI:** 10.3389/fphar.2020.585096

**Published:** 2020-10-08

**Authors:** Cristoforo Silvestri, Ester Pagano, Sébastien Lacroix, Tommaso Venneri, Claudia Cristiano, Antonio Calignano, Olga A. Parisi, Angelo A. Izzo, Vincenzo Di Marzo, Francesca Borrelli

**Affiliations:** ^1^Centre de recherche de l’Institut universitaire de cardiologie et de pneumologie de Québec (IUCPQ), Québec, QC, Canada; ^2^Département de médecine, Faculté de Médecine, Université Laval, Québec, QC, Canada; ^3^Department of Pharmacy, School of Medicine and Surgery, University of Naples Federico II, Naples, Italy; ^4^Institut sur la nutrition et les aliments fonctionnels (INAF), Québec, QC, Canada; ^5^Institute of Biomolecular Chemistry, National Research Council (CNR) of Italy, Pozzuoli, Italy; ^6^Centre Nutriss, École de nutrition, Faculté des sciences de l’agriculture et de l’alimentation (FSAA), Université Laval, Québec, QC, Canada; ^7^Joint International Unit between the National Research Council (CNR) of Italy and Université Laval on Chemical and Biomolecular Research on the Microbiome and its Impact on Metabolic Health and Nutrition (UMI-MicroMeNu), Institute of Biomolecular Chemistry, CNR, Pozzuoli, Italy; ^8^Canada Research Excellence Chair on the Microbiome-Endocannabinoidome Axis in Metabolic Health (CERC-MEND), Université Laval, Québec, QC, Canada

**Keywords:** colitis, cannabinoid, gut-brain axis, fish oil, microbiome

## Abstract

Inflammatory bowel disorders can be associated with alterations in gut microbiota (dysbiosis) and behavioral disturbances. In experimental colitis, administration of fish oil (FO) or cannabinoids, such as cannabidiol (CBD), reduce inflammation. We investigated the effect of combined FO/CBD administration on inflammation and dysbiosis in the dextran sulphate sodium (DSS) model of mouse colitis, which also causes behavioral disturbances. Colitis was induced in CD1 mice by 4% w/v DSS in drinking water for five consecutive days followed by normal drinking water. FO (20–75 mg/mouse) was administered once a day starting two days after DSS, whereas CBD (0.3–30 mg/kg), alone or after FO administration, was administered once a day starting 3 days after DSS, until day 8 (d8) or day 14 (d14). Inflammation was assessed at d8 and d14 (resolution phase; RP) by measuring the Disease Activity Index (DAI) score, change in body weight, colon weight/length ratio, myeloperoxidase activity and colonic interleukin (IL)-1β (IL-1β), IL-10, and IL-6 concentrations. Intestinal permeability was measured with the fluorescein isothiocyanate-dextran. Behavioral tests (novel object recognition (NOR) and light/dark box test) were performed at d8. Fecal microbiota composition was determined by ribosomal 16S DNA sequencing of faecal pellets at d8 and d14. DSS-induced inflammation was stronger at d8 and accompanied by anxiety-like behavior and impaired recognition memory. FO (35, 50, 75 mg/mouse) alone reduced inflammation at d8, whereas CBD alone produced no effect at any of the doses tested; however, when CBD (3, 10 mg/kg) was co-administered with FO (75 mg/mouse) inflammation was attenuated. FO (20 mg/mouse) and CBD (1 mg/kg) were ineffective when given alone, but when co-administered reduced all inflammatory markers and the increased intestinal permeability at both d8 and d14, but not the behavioral impairments. FO, CBD, and their combination affected gut bacteria taxa that were not affected by DSS *per se*. *Akkermansia muciniphila*, a species suggested to afford anti-inflammatory action in colitis, was increased by DSS only at d14, but its levels were significantly elevated by all treatments at d8. FO and CBD co-administered at *per se* ineffective doses reduce colon inflammation, in a manner potentially strengthened by their independent elevation of *Akkermansia muciniphila*.

## Introduction

Ulcerative colitis (UC) and Crohn’s disease (CD), the two most common inflammatory bowel diseases (IBDs), are chronic, relapsing, and lifelong ailments characterized by strong inflammation of the colon. They affect millions of people worldwide with increasing incidence (Kaplan and Ng, 2017; Ng et al., 2018). IBDs result from the interaction between environmental, genetic, and epigenetic risk factors causing an excessive immune response in the mucosa leading to uncontrolled inflammation, and represent, in turn, a risk factor for the development of colorectal cancer (Keller et al., 2019). Recent evidence suggests that the imbalance of the gut microbiota ecosystem, also known as gut dysbiosis, is linked to the initiation and progression of IBDs (De Musis et al., 2020). In fact, dysbiosis, through disruption of the intestinal epithelial barrier and ensuing entry of gram-negative bacteria-derived pro-inflammatory molecules such as lipopolysaccharide (LPS) into the blood stream may contribute to systemic inflammation. However, it is still unclear whether gut dysbiosis is one of the primary causes of IBD, or if it is secondary to IBD-induced mucosal inflammation and exacerbates its consequences (Ananthakrishnan et al., 2018; Pittayanon et al., 2020).

Fish oil (FO), mostly thanks to its high content in omega-3 polyunsaturated fatty acids (n-3-PUFAs), i.e., eicosapentaenoic (EPA; C20:5) and docosahexaenoic (DHA; C22:6) fatty acids, has been suggested to produce important anti-inflammatory actions both in pre-clinical and clinical studies [see (Calder, 2017) for review]. Several mechanisms have been proposed for this property of FO, including, but not limited to, the capability of EPA and DHA to: 1) replace arachidonic acid (AA) in membrane phospholipids, and hence reduce the amounts of this omega-6 PUFA that can act as direct or indirect biosynthetic precursors for endocannabinoids and pro-inflammatory eicosanoids (Calder, 2017; Innes and Calder, 2018); and 2) affect the gut microbiota to ameliorate gut dysbiosis and counteract, among others, its contribution to chronic, lowgrade inflammation (Costantini et al., 2017). As a consequence, it has been suggested that FO may provide therapeutic relief for IBDs (Marton et al., 2019). Also, plant cannabinoids from *Cannabis sativa*, and in particular: 1) Δ^9^-tetrahydrocannabinol (THC), which activates cannabinoid receptor of type-1 (CB1) or, particularly, type-2 (CB2) [see (Ibeas Bih et al., 2015; Turner et al., 2017) for review], and 2) cannabidiol (CBD), which modulates several pro-inflammatory targets [see (Burstein, 2015) for review] have been shown to produce anti-inflammatory effects in animal models of several inflammatory disorders, including IBDs (Gotfried et al., 2020; Williamson et al., 2020). Importantly, purified plant-derived CBD is now currently approved in both the USA (as Epidiolex^®^) and Europe (as Epidyolex^®^, as an adjunctive therapy with clobazam) as an effective treatment for seizures associated with Dravet syndrome and Lennox-Gastaut syndrome (intractable rare pediatric epilepsies); while generally well tolerated, diarrhea is a common adverse event (Pauli et al., 2020).

We have shown that, in the dinitrobenzenesulphonic acid (DNBS) and croton oil models of lower and upper intestinal inflammation, CBD can produce beneficial effects, although often with lower potency/efficacy than other cannabinoids (Borrelli et al., 2009; Borrelli et al., 2013; Romano et al., 2013; Pagano et al., 2019) or CBD-enriched *Cannabis* extracts (Pagano et al., 2016). A CBD-rich extract was indeed tested in an open label phase II trial against UC and, although promising results were seen on some secondary endpoints (subjective physician’s global assessment of illness severity, subject global impression of change and patient-reported quality-of-life outcomes), it did not achieve statistically significant results for the primary endpoint (percentage of patients in remission after treatment) (Irving et al., 2018). Given the potential advantages afforded by the use in IBDs of a drug already approved for other indications, we hypothesized that a possible way to improve CBD efficacy and potency at counteracting inflammation would be through its oral co-administration with dietary FO. To test this, we developed a co-administration protocol of the two treatments in a widely used animal model of IBD, and UC in particular, i.e., the dextran sulphate sodium (DSS)-induced colitis in mice. This model has been described to: 1) produce effects on the gut microbiota composition that could contribute to colonic inflammation, and hence has been employed to investigate the anti-inflammatory potential of treatments targeting gut dysbiosis (Munyaka et al., 2016; Ke et al., 2020; Liu et al., 2020); and 2) be accompanied by behavioral cognitive and affective impairments (Reichmann et al., 2015), which, in view of the ever increasing evidence in favor of the microbiota-gut-brain axis [see (Emge et al., 2016) for review], could also be the consequence of DSS-induced dysbiosis. We co-administered different oral doses of FO and CBD during the development of colonic inflammation by administration of DSS until either its peak, at day 8 (d8), or the inflammatory resolution phase (RP), at d14. We also investigated if the potentially stronger effects of combined FO and CBD treatment was accompanied by effects on DSS-induced gut dysbiosis at d8 and d14, or on behavioral impairments at d8.

## Materials and Methods

### Drugs and Reagents

Dextran Sulfate Sodium (DSS, molecular weight 36,000–50,000) and myeloperoxidase (MPO) from human leucocytes were purchased from MP Biomedical (Illkirch, France) and Sigma Aldrich S.r.l. (Milan, Italy), respectively. Purified, botanically derived CBD (≥98%), was supplied by GW Research Ltd (Cambridge, UK). FO (Marco Viti Farmaceutici S.p.A (Mozzate, Como, Italy)] and sesame oil [SO, Il fiore di Loto S.r.l. (Orbassano, Torino, Italy)] were obtained from a local pharmacy. All chemicals and reagents employed in this study were of analytical grade. CBD was dissolved in sesame oil (90 µl/mouse). Sesame oil had no significant effects on the responses under study.

### Animals

Male CD1 mice (weighing 28–30 g) were obtained from Charles River Laboratories (Calco, Lecco, Italy) and housed per experimental group in polycarbonate cages (Tecniplast S.p.A), under a 12-h light/dark cycle, controlled temperature (23 ± 2°C) and constant humidity (60%). Mice had free access to tap water and standard rodent diet (Mucedola srl, Milan, Italy). All mice were fasted 2 h before the oral gavage of CBD and FO. Mice were randomly allocated to different experimental groups (at least six animals for each group, five to six for each cage) of equal size and outcome assessments were performed in blind. All the experimental protocols were evaluated and approved by the Institutional Animal Ethics Committee for the use of experimental animals and conformed to guidelines for the safe use and care of experimental animals in accordance with the Italian D.L. no. 116 of 27 January 1992 and associated guidelines in the European Communities Council (86/609/ECC and 2010/63/UE). Animal studies are reported in compliance with the ARRIVE guidelines (Kilkenny et al., 2010; McGrath and Lilley, 2015) and with the recommendations made by the *British Journal of Pharmacology* (Curtis et al., 2018). G Power was used for sample size calculation (Faul et al., 2007).

### Induction of Murine Colitis and Pharmacological Treatments

**Figure 1:**
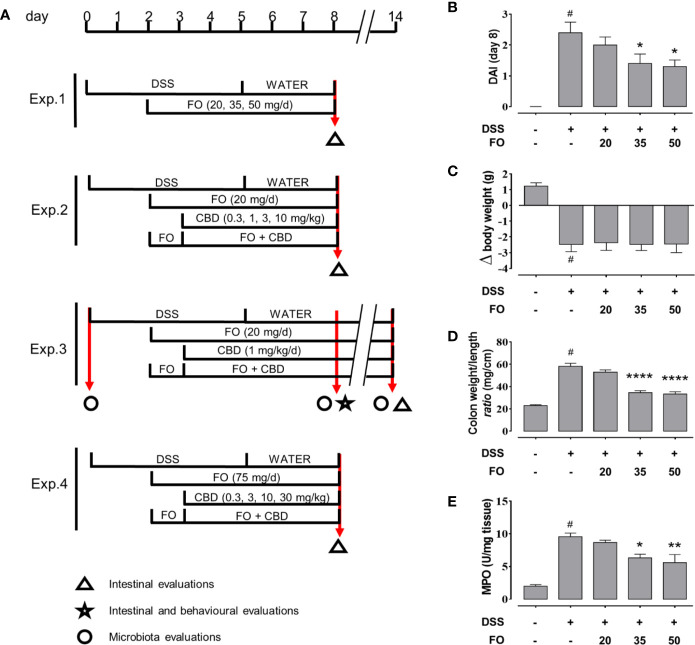
**(A)** Experimental protocols and time points of intestinal, inflammation and microbiota evaluations. **(B–E)** Fish oil (FO) reduces inflammation in a dose-dependent manner in DSS-treated mice (Experiment 1). Effect of FO (20, 35, and 50 mg/mouse, by oral gavage) on disease activity index (DAI) score **(B)**, colon weight/colon length ratio **(C)**, MPO activity **(D)**, and body weight **(E)**, in DSS-treated mice (weighing 28–30 g). Bars are mean ± SEM of 10 animals for **(B, C, E)** and five tissues for **(D)** for each experimental group. Data in **(B)** [F_(4,45)_ = 12.94; p < 0.0001], **(C)** [F_(4,45)_ = 14.91; p < 0.0001], **(D)** [F_(4,20)_ = 18.95; p < 0.0001], and **(E)** [F_(4,45)_ = 57.33; p < 0.0001] were statistically analyzed using one-way ANOVA followed by the Dunnett’s multiple comparisons test ^#^p < 0.0001 vs. control; *p < 0.05, **p < 0.01, and ****p < 0.0001 vs. DSS. V, vehicle (sesame oil).

Colitis was induced in CD1 mice by providing 4% w/v DSS in drinking water for five consecutive days followed by normal drinking water for another 3 or 9 days (DSS water was changed every 2 days) (Chassaing et al., 2014). FO was administered once a day starting two days after DSS and continued every day until d8 or d14. CBD, dissolved in sesame oil, was administered once a day starting one day after the administration of FO (i.e., 3 days after DSS), in order not to overstress the mice with two administrations in the initial phase of the pro-inflammatory treatment and continued every day until euthanasia on d8 or d14 (see **Figure 1A**). Additionally, in preliminary experiments FO was given either simultaneously or one day before CBD; results showed a greater beneficial effect of CBD when it was given one day after FO (data not shown). Therefore, all the experiments were carried out according to this schedule of administration. All animals were euthanized by asphyxiation with CO2.

Four experiments were carried out (**Figure 1A**): Experiment 1) a dose-response curve for FO (20–50 mg/mouse each mouse weighing 28–30 g), to find the highest inactive dose of FO; Experiment 2) a dose-response curve for CBD (0.3–10 mg/kg by gavage in sesame oil) in the presence or absence of FO (20 mg/mouse), to find the highest inactive dose of CBD; Experiment 3) a co-administration of an inactive dose of FO (20 mg/mouse) with an inactive dose of CBD (1 mg/kg), to assess the potential stronger anti-inflammatory effects of a combination treatment in the acute and remission phases of DSS-induced colitis; and Experiment 4) a co-administration of FO at a dose of 75 mg/mouse with different doses (0.3–30 mg/kg) of CBD, to determine even potentially stronger effects on markers of inflammation through the combination of non-inactive doses. In the first, second and fourth experiments, animals were sacrificed at d8, and in the third at both d8 (immediately after behavioral tests) and d14 (RP), (see **Figure 1**). In the third set of experiments, intestinal inflammatory parameters were evaluated at both d8 and d14, whereas behavioral tests were performed at d8 only. In the other experiments, only intestinal inflammatory parameters were evaluated at d8 (**Figure 1**). Stools were collected within 1 h directly from mice previously kept in clean separate cages at d0, d8 and d14 (immediately before the sacrifice) and quickly stored at −80°C. The results of each experimental group were pooled.

For Experiment 1, animals were divided into the following groups: (1) Control (no vehicle or treatment); (2) DSS; (3) DSS + FO 20 mg/mouse; (4) DSS + FO 35 mg/mouse; (5) DSS + FO 50 mg/mouse.

For Experiment 2, animals were divided into the following groups: (1) Control (no vehicle or treatment); (2) DSS; (3) DSS + vehicle (sesame oil, 90 µl/mouse); (4) DSS+ FO 20 mg/mouse; (5) DSS+ CBD 0.3 mg/kg; (6) DSS+ CBD 1 mg/kg; (7) DSS+ CBD 3 mg/kg; (8) DSS+ CBD 10 mg/kg; (9) DSS+ FO 20 mg/mouse+ CBD 0.3 mg/kg; (10) DSS+ FO 20 mg/mouse+ CBD 1 mg/kg; (11) DSS+ FO 20 mg/mouse+ CBD 3 mg/kg; (12) DSS+ FO 20 mg/mouse+ CBD 10 mg/kg.

For Experiment 3, animals were divided into the following groups: (1) Control (no vehicle or treatment) (2) vehicle (sesame oil, 90 µl/mouse); (3) FO 20 mg/mouse; (4) CBD 1 mg/kg; (5) CBD + 1 mg/kg +FO 20 mg/mouse; (6) DSS; (7) DSS + vehicle; (8) DSS + FO 20 mg/mouse; (9) DSS+ 1 mg/kg CBD; (10) DSS+ 20 mg/mouse FO + 1 mg/kg CBD.

For Experiment 4, animals were divided into the following groups: (1) Control (no vehicle or treatment); (2) DSS; (3) DSS + vehicle (sesame oil, 90 µl/mouse); (4) DSS + FO 75 mg/mouse; (5) DSS + CBD 0.3 mg/kg; (6) DSS + CBD 3 mg/kg; (7) DSS + CBD 10 mg/kg; (8) DSS + CBD 30 mg/kg; (9) DSS+ FO 75 mg/mouse + CBD 0.3 mg/kg; (10) DSS+ 75 mg/mouse+ 75 mg/mouse FO+ CBD 3 mg/kg; (11) DSS+ 75 mg/mouse FO+ CBD 10 mg/kg; (12) DSS + 75 mg/mouse FO + CBD 30 mg/kg.

### Assessment of Colitis

Body weight, food, and water consumption were measured daily throughout the experiment. Stool consistency and visible blood in faeces were also examined to determine the Disease Activity Index (DAI) score [**Table 1**, (Nishiyama et al., 2012)]. At the time of sacrifice, colons were removed and colon weight/colon length *ratio* was measured. Colons were then snap frozen at −80°C for determination of myeloperoxidase (MPO) activity and interleukin (IL) levels.

**Table 1:** Disease activity index (DAI) scoring system [adapted from (Nishiyama et al., 2012)].

Score	Diarrheal stool score	Bloody stool score
**0**	Normal stool	Normal colored stool
**1**	Midly soft stool	Brown stool
**2**	Very soft stool	Reddish stool
**3**	Watery stool	Bloody stool

The sum of the scores of two parameters was defined as the DAI score.

### Myeloperoxidase Activity

MPO activity, a marker used to quantify the extent of neutrophil accumulation in whole-tissue colons (Krawisz et al., 1984), was determined in full-thickness colons. Briefly, tissues were homogenized in an appropriate lysis buffer composed of 0.5% hexadecyltrimethylammonium bromide in 3-(N-morpholino)propanesulfonic acid (MOPS) 10 mM in a ratio of 50 mg tissue/1 ml MOPS. The samples were then centrifuged for 20 min at 15,000 x g at 4°C. An aliquot of the supernatant was then incubated with NaPB (sodium phosphate buffer pH 5.5) and 3,3′,5,5′-tetramethylbenzidine (16 mM). After 5 min, H_2_O_2_ (1mM) in NaPB was added and the reaction stopped by adding acetic acid. The rate of change in absorbance was measured by a spectrophotometer at 650 nm. Different dilutions of human MPO enzyme of known concentration were used to obtain a standard curve (representative R^2^ = 0.94). MPO activity was expressed as unit(U)/mg of tissue.

### Interleukin Levels Determination

Interleukin (IL)-1β (IL-1β), IL-10, and IL-6 concentrations were determined in homogenates obtained from full-thickness mice colonic tissues using commercial ELISA kits (ThermoFisher Scientific, Milano) according to manufacturer’s instructions.

### Intestinal Permeability Assay

Intestinal permeability was examined using a fluorescein isothiocyanate (FITC)-labelled-dextran method (Pagano et al., 2019). Briefly, the day before sacrifice (day 7 and day 13), mice received fluorescein isothiocyanate (FITC)-conjugated dextran (molecular mass 3–5 kDa; 600 mg/kg) by oral gavage. One day later, blood was collected by cardiac puncture, and the serum was immediately analyzed for FITC-derived fluorescence using a fluorescent microplate reader (excitation-emission wavelengths: 485–520 nm). Serially diluted FITC-dextran was used to generate a standard curve. Intestinal permeability was expressed as nM FITC found in the serum.

### Behavioral Tests

Behavioral tests: novel object recognition (NOR) task to evaluate recognition memory and Light/Dark box test to evaluate anxiety (Guida et al., 2017; Zhu et al., 2018) were performed at d8 only.

NOR task was done as described previously with some modifications (Zhu et al., 2018). Mice were placed on at a time in a cage (40 cm × 25 cm × 18 cm) in the presence of two identical objects (training phase) and filmed for 10 min. Successively, one object was replaced with a new object and mice were placed again in the cage for 10 min (testing phase). The tests were automatically detected by a video camera coupled with video-tracking software (Any-maze, Stoelting Co., Wood Dale, IL, USA). Video clips were analyzed considering the number of explorations of the new object and the meters traveled in the cage (spontaneous locomotion). Mice with cognition disorders spend less time with the new object. After each trial, the cages and the objects were cleaned with 70% ethanol in order to remove odor cues.

Light/Dark box was performed as previously reported (Guida et al., 2017). Mice, one at a time, were placed for 10 min in a light and dark box apparatus, i.e., a box (60 cm × 30 cm × 30 cm) divided in a dark area and a light area (equally sized compartments, 30 x 30 cm each). Mice were placed in the light area and allowed to move freely. Time spent in dark side (mice with anxiety spent more time in dark) and number of transitions between light side and dark side (in order to observe mouse movements) were evaluated.

### Statistical Analysis

The data and statistical analysis comply with the British Journal of Pharmacology’s recommendations and requirements on experimental design and analysis (Curtis et al., 2018). Results are expressed as mean ± SEM. Data were analyzed for normality using the Anderson-Darling method (http://www.kevinotto.com/RSS/templates/Anderson-DarlingNormalityTestCalculator.xls). Group comparisons were assessed using one-way ANOVA (followed by the Dunnett’s or Tukey-Kramer multiple comparisons test). *Post-hoc* tests were conducted only if F achieved P < 0.05 and there was no significant variance in homogeneity. Analysis was performed using GraphPad Prism 7.00 (La Jolla, USA). According to recent preclinical guidelines in pharmacology, statistical analysis was undertaken only when each group size (i.e., number of independent values) had a minimum of n = 5 independent animals/samples. Statistical analysis was performed using independent values and technical replicates were not considered independent values. A P value less than 0.05 was considered significant.

### Analysis of the Faecal Microbiome

DNA was extracted from faeces using the QIAmp PowerFecal DNA kit (Qiagen, Hilden, Germany) according to the manufacturers’ instructions. The DNA concentrations of the extracts were measured fluorometrically with the Quant-iT PicoGreen dsDNA Kit (Thermo Fisher Scientific, MA, USA) and the DNAs were stored at −20°C until 16S rDNA library preparation. Briefly, 1 ng of DNA was used as template and the V3-V4 region of the 16S rRNA gene was amplified by polymerase chain reaction (PCR) using the QIAseq 16S Region Panel protocol in conjunction with the QIAseq 16S/ITS 384-Index I (Sets A, B, C, D) kit (Qiagen, Hilden, Germany) (Rausch et al., 2019). The 16S metagenomic libraries were eluted in 30 µl of nuclease-free water and 1 µl was qualified with a Bioanalyzer DNA 1000 Chip (Agilent, CA, USA) to verify the amplicon size (expected size ~600 bp) and quantified with a Qubit (Thermo Fisher Scientific, MA, USA). Libraries were then normalized and pooled to 2 nM, denatured and diluted to a final concentration of 6 pM and supplemented with 5% PhiX control (Illumina, CA, USA). Sequencing (2 × 275 bp paired-end) was performed using the MiSeq Reagent Kit V3 (600 cycles) on an Illumina MiSeq System. Sequencing reads were generated in less than 65 h. Image analysis and base calling were carried out directly on the MiSeq. Data was processed using the DADA2 pipeline and taxonomic assignation with reference to the RDP database (Callahan et al., 2016). All sequences were cumulative sum scaled (CSS) (Paulson et al., 2013).

### Statistical Analysis of Faecal Microbiome Data

The primary objective of the analysis was to evaluate the impact of DSS-induced colitis on gut microbiota composition in comparison to control and to evaluate the potential that CBD, FO or a combination of both have on reversing the DSS-associated disturbances. Vehicle (sesame oil) treated mice were used as control for DSS-Vehicle, and the latter as a control for DSS-CBD, DSS-FO, or DSS-CBD + FO.

Outliers were defined as samples outside the 95% CI ellipse by a PCA. Following this analysis, no samples were defined as outliers and analyses were therefore carried on all samples of interest.

The *Firmicutes* to *Bacteroidetes* ratio is often, but not always, positively associated to diet-induced obesity and dysmetabolism as well as other inflammatory conditions (Ley et al., 2006; Turnbaugh et al., 2008; Boulange et al., 2016; Forbes et al., 2016; Santoru et al., 2017). Asterisks (if none: ns) displayed above boxes represent Kruskal Wallis p-values * p < 0.05. Wilcoxon p-values for pairwise comparisons (within group) are displayed above brackets.

The heatmap.2 package for R was used to represent bacterial family composition between treatment groups and time points using CSS-normalized bacterial counts. Bacterial families or treatment groups and time points were clustered using unsupervised hierarchical clustering.

Differential abundance testing was assessed using two-way ANOVA (taxa ~ Group*Day) and Tukey HSD *post-hoc* p-values. Data is represented in box plots with boxes showing first, second and third quartiles and whiskers indicating samples within 1.5 times the interquartile range. Samples outside this range are indicated by dots.

## Results

### FO Reduces Inflammatory Colitis in Mice in a Dose Dependent Manner (Experiment 1)

FO, administered at 20, 35, or 50 mg/mouse (by oral gavage) to DSS-treated mice, significantly attenuated the DAI score (**Figure 1B**), colon weight/colon length ratio (**Figure 1C**) and MPO activity (**Figure 1D**), in a dose-dependent manner. Despite this, DSS-induced loss of body weight was unaffected by any FO dose (**Figure 1E**).

### Combined Administration of FO and CBD at *Per Se* Ineffective Doses Is Associated With Reduced Colon Inflammation in DSS-Treated Mice (Experiment 2)

**Figure 2:**
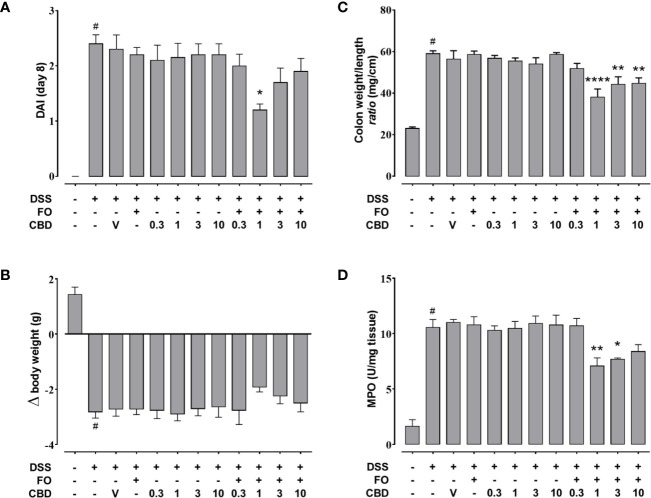
Effect of fish oil (20 mg/mouse) and CBD (0.3–10 mg/kg), both alone and in combination, on intestinal inflammation in DSS-treated mice (Experiment 2). Effect of sesame oil (V, 90 µl/mouse, by oral gavage, used as a vehicle control), fish oil [FO, 20 mg/mouse, by oral gavage], CBD (0.3–10 mg/kg, by oral gavage) and FO + CBD on disease activity index (DAI) score **(A)**, body weight **(B)**, colon weight/colon length ratio **(C)**, and MPO activity **(D)** in DSS-treated mice (weighting 28–30 g). On the *x*-axis the doses shown are for CBD. Bars are mean ± SEM of 10 animals **(A–C)** or of five tissues **(D)** for each experimental group. Data in **(A)** [F_(11,108)_=10.41; p < 0.0001], **(B)** [F_(11,108)_ = 16.14; p < 0.0001], **(C)** [F_(11,108)_=18.3; p < 0.0001], and **(D)** [F_(11,48)_ = 19.36; p < 0.0001] were statistically analyzed using one-way ANOVA followed by the Dunnett’s multiple comparisons test ^#^p < 0.0001 vs. control, *p < 0.05, **p < 0.01, and ****p < 0.0001 vs. DSS + FO.

CBD, given by oral gavage at the dose range of 0.3-10 mg/kg did not affect DSS-induced intestinal inflammation for any of the four endpoints measured (**Figure 2**). However, co-administration of a *per se* ineffective dose of FO (20 mg/mouse; **Figure 1**) with CBD reduced DAI score (**Figure 2A**), colon weight/colon length ratio (**Figure 2C**) and MPO activity (**Figure 2D**), but not the loss of body weight (**Figure 2B**) in DSS-treated mice; a numerical reduction was observed for the latter with CBD 1 mg/kg but this did not reach significant levels. While the effects appeared to be consistent across all concentrations above CBD 1 mg/kg, this was the most effective dose and was therefore, selected for Experiment 3.

### Co-Administered *Per Se* Ineffective Doses of FO and CBD Reduce Colon Inflammation and Epithelial Barrier Permeability in DSS-Treated Mice, but Do Not Affect Behavioral Impairments (Experiment 3)

**Figure 3:**
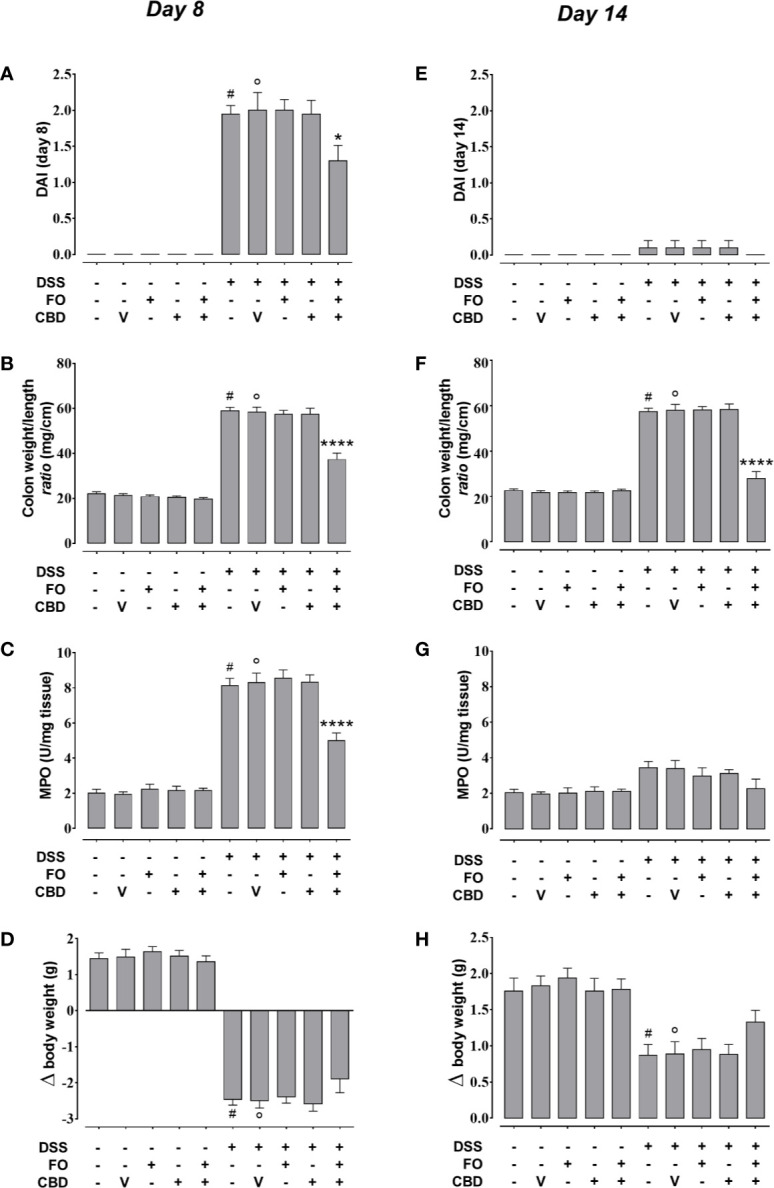
Effect of fish oil (20 mg/mouse) and CBD (1 mg/kg), both alone and in combination, on intestinal inflammation at its peak (day 8) and during remission (day 14) in DSS-treated mice (Experiment 3). Effect of sesame oil (V, 90 µl/mouse, by oral gavage, used as CBD vehicle), fish oil (FO, 20 mg/mouse, by oral gavage), CBD (1 mg/kg, by oral gavage) and FO + CBD on disease activity index (DAI) score **(A, E)**, weight/colon length ratio (B,F), MPO activity (C,G) and body weight (D,H) in control mice (without DSS treatment) and in animals with colitis (induced by DSS) at day 8 **(A–D)** or day 14 **(E–H)** from the first injection of DSS. Bars are mean ± SEM of 10 animals **(A, B, D–F, H)** or five tissues **(C, G)** for each experimental group. Data in **(A)** [F_(9,90)_ = 54.88; p < 0.0001], **(B)** [F_(9,90)_ = 121.3; p < 0.0001], **(C)** [F_(9,40)_ = 84.75; p < 0.0001], **(D)** [F_(9,90)_=98.41; p < 0.0001], **(E)** [F_(9,90)_ = 0.653; p = 0.7485], **(F)** [F_(9,90)_ = 109.9; p < 0.0001], **(G)** [F_(9,40)_ = 3.277; p = 0.0045], and **(H)** [F_(9,90)_ = 8.823; p < 0.0001] were statistically analyzed using one-way ANOVA followed by the Tukey-Kramer multiple comparisons test ^#^p < 0.0001 **(A–D, F)** or p < 0.01 **(H)** vs. control; °p < 0.0001 **(A–D, F)** or p < 0.01 **(H)** vs. vehicle; *p < 0.05 and ****p < 0.0001 vs. DSS + vehicle and DSS + FO and DSS + CBD.

**Figure 4:**
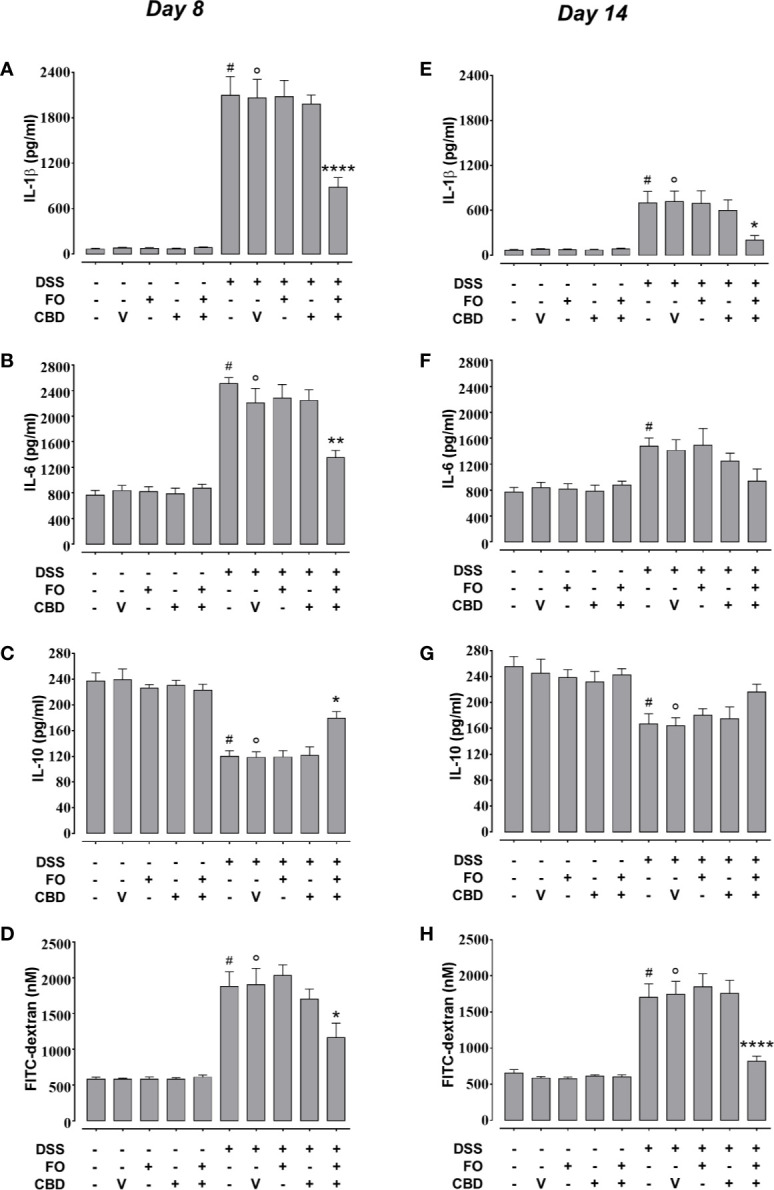
Effect of fish oil (20 mg/mouse) and CBD (1 mg/kg), both alone and in combination, on DSS-induced changes in interleukin-1β, interleukin-6, and interleukin-10 levels and intestinal permiability (Experiment 3). Effect of sesame oil (V, 90 µl/mouse, by oral gavage, used as a control), fish oil (FO, 20 mg/mouse, by oral gavage), CBD (1 mg/kg, by oral gavage) and FO + CBD on interleukin-1β **(A, E)**, interleukin-6 **(B, F)**, interleukin-10 **(C, G)** and serum FICT-dextran concentration (a measure of intestinal barrier function; **D, H**) in control and DSS-treated mice (weighing 28–30 g) at day 8 **(A–D)** and day 14 **(E–H)** from DSS injection. Bars are mean ± SEM of five tissues **(A–D, E, F)** or 6 serum samples **(D, H)** for each experimental group. Data in **(A)** [F_(9,40)_ = 48.44; p < 0.0001], **(B)** [F_(9,40)_ = 31.52; p < 0.0001], **(C)** [F_(9,40)_ = 25.34; p < 0.0001], **(D)** [F_(9,50)_ = 22.99; p < 0.0001], **(E)** [F_(9,40)_ = 9.521; p < 0.0001], (**F**) [F_(9,40)_ = 4.837; p = 0.0002], **(G)** [F_(9,40)_ = 5.822; p < 0.0001], and **(H)** [F_(9,50)_ = 23.78; p < 0.0001] were statistically analyzed using one-way ANOVA followed by the Tukey-Kramer multiple comparisons test. ^#^p < 0.05 **(F)** or p < 0.01 **(E, G)** or p < 0.0001 vs. control **(A–D, H)**; °p < 0.05 **(G)**, 0.001 **(E)**, or p < 0.0001 **(A–D, H)** vs. vehicle; *p < 0.05, **p < 0.01, and ****p < 0.0001 vs. DSS + vehicle or DSS + FO or DSS + CBD.

We confirmed the results of experiments 1 and 2 that FO (20 mg/mouse), CBD (1 mg/kg) or CBD vehicle (sesame oil’ SO), all given alone by oral gavage, did not affect DSS-induced intestinal inflammation (**Figure 3**). However, combined treatment with CBD (1 mg/kg) and FO (20 mg/mouse) significantly reduced the changes induced by DSS administration at d8 (corresponding to active disease phase) on DAI score (**Figure 3A**), colon weight/colon length ratio (**Figure 3B**), and MPO activity (**Figure 3C**), but there was no effect on the loss of mice body weight (**Figure 3D**). Some of these effects were also present at the RP (d14), when the overall degree of inflammation was lower than at d8 (**Figures 3E–H**), and the combination restored almost all intestinal parameters to control levels. When given in combination, but not alone, CBD and FO also significantly reduced DSS-induced increases in intestinal permeability at d8 and d14, and increases in IL-6 and IL-1β and decreases in IL-10 levels at d8. At d14 the combined treatment only reduced the DSS-induced increase of IL-1β levels (**Figure 4**).

CBD (1 mg/kg) and FO (20 mg/mouse), either given alone or in combination, did not affect the DSS-induced behavioral changes in the light-dark box (anxiety test) and NOR (cognitive ability test) at d8 (**Supplementary Figure 1**).

### *Per Se* Ineffective Doses of FO and CBD, Alone or in Combination, Produce Profound Effects on the Gut Microbiome of DSS-Treated Mice (Experiment 3)

**Figure 5:**
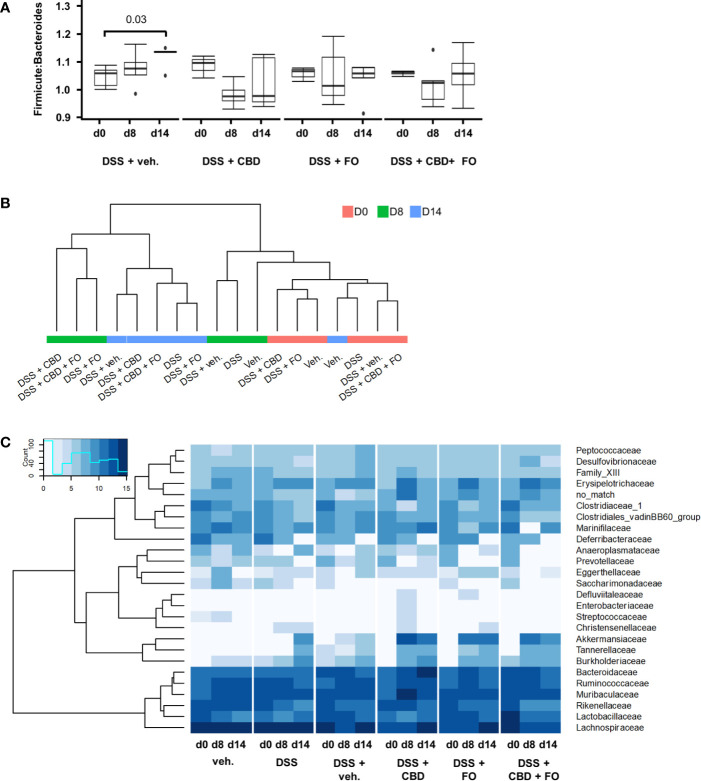
Effect of fish oil (20 mg/mouse) and CBD (1 mg/kg), both alone and in combination, both at day 8 and day 14, on microbiota in faecal samples collected from DSS-treated mice belonging to the same treatment groups as – (Experiment 3). **(A)** Firmicutes:Bacteroidetes ratio. Wilcoxon P-values for pairwise comparisons are displayed above brackets. **(B)** Hierarchical clustering of treatment groups using CSS-normalized bacterial family counts. **(C)** Heat map and hierarchical clustering of family composition using CSS-normalized bacterial counts.

Neither DSS nor the treatments or their combination with DSS affected in a statistically significant manner Shannon diversity of mouse fecal microbiome (data not shown). At the level of phyla, the Firmicutes:Bacteroidetes ratio increased with time after DSS, but significantly only at the RP (d14). All treatments (i.e., CBD, FO, and CBD + FO) prevented this time-related increase (**Figure 5A**). No statistically significant difference among treatments was observed at either d8 or d14.

Hierarchical clustering of sequencing counts at the family level revealed that all d0 and vehicle-alone groups (irrespective of day) clustered together as did the DSS and DSS + Veh groups at d8 (i.e., during the inflammatory peak; **Figure 5B**). Most notably, all DSS-treated groups at d14 (i.e., the RP) clustered together independently but within a larger cluster than included the experimental treatments (CBD, FO, and CBD + FO) at d8.

**Figure 6:**
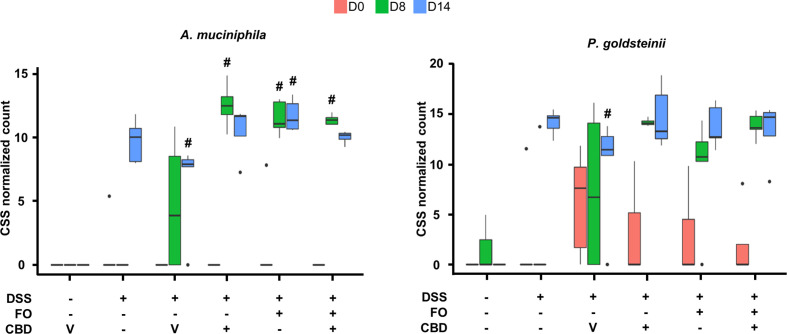
Effect of fish oil (20 mg/mouse) and CBD (1 mg/kg), both alone and in combination, both at day 8 and day 14, on the relative abundance of *Akkermansia muciniphila* and *Parabacteroides goldsteinii* in faecal samples collected from DSS-treated mice belonging to the same treatments groups as – (Experiment 3). Only the species for which statistically significant differences were observed between DSS-Veh and Veh at day 14 are shown. Data were analyzed by two-way ANOVA followed by Tukey HSD *post-hoc* tests #, P < 0.05 vs. relevant control of the same day. For F values, please see **Supplementary Tables 1**.

DSS treatment (DSS + Veh vs. Veh) affected several families, genera and species of gut bacteria mostly at RP (d14) (**Figures 5C**, **6** and **Supplementary Figures 2**–**5**; see **Supplementary Tables 1**–**3** for statistical details).

The only families that showed numerical alterations without reaching statistical differences at d8 were *Saccharimonadaceae* (P = 0.06) and *Streptococcaceae* (P = 0.096), which were reduced, with the latter family being increased by CBD (DSS + CBD vs. DSS + Veh, **Figure 5C** and **Supplementary Figure 2**). On the other hand, at RP (d14), *Akkermansiaceae* and *Tannerellaceae* were increased in the DSS + Veh vs. Veh group, and this increase was significant also at d14 with all three treatments and clustered together within the heatmap in which family sequencing counts were subjected to hierarchical clustering (**Figure 5C** and **Supplementary Figure 2**).

Several families that were not modified by DSS, were instead modified from their relative abundancies in DSS + Veh mice by one or more of the three treatments at either d8 or d14. These included: *Clostridiaceae*_1 (reduced by CBD at d8), *Defluviitaleaceae* (increased by CBD and FO at d8), *Marinifilaceae* (decreased by CBD + FO at d8), *Christensenellaceae* (increased in a statistically significant manner by FO at d14 but not d8), *Desulfovibrionaceae* (decreased by CBD + FO at d14). A numerical decrease, which did not reach statistical significance (P = 0.1), was also seen at d14 with *Ruminococcaceae* in the presence of CBD + FO (**Supplementary Figure 3**).

At the genus level (**Supplementary Figure 4**), the only taxa for which a numerical increase, which did not reach statistical significance, was observed at d8 following DSS was *Akkermansia* (P = 0.09). This increase was however, further increased in a statistically significant manner by all treatment groups at d8. *Akkermansia* was increased by DSS at d14, and as for d8 all treatments resulted in even greater increases. The genus *Acetitomaculum*, instead, was significantly decreased only at d14, and none of the treatments could reverse this effect (**Supplementary Figure 4**). There were, however, several genera that were not affected by DSS but were significantly different from DSS + Veh following treatments (**Supplementary Figure 5**): *Anaerotruncus* and *Candidatus*_*Arthromitus* were both decreased at d8 by CBD, whereas, at the same time point, *Odoribacter* was decreased by CBD + FO, and *Defluviitaleaceae-UCG011* was increased by CBD and FO. On the other hand, at d14, *Christensenellaceae_R7-group* was increased by FO, *Ruminococcaceae-UCG-005* was decreased by CBD, and *Tyzzerella_3* was decreased by FO and CBD + FO.

Finally, although the method used does not usually allow identification of species, we could identify *Akkermansia muciniphila* and *Parabacteroides goldsteinii* as being increased by DSS at d14, and, in the presence of all treatments, also at d8 (**Figure 6**).

### An Effective Dose of FO Administered Together With CBD Abolishes Colon Inflammation (Experiment 4)

**Figure 7:**
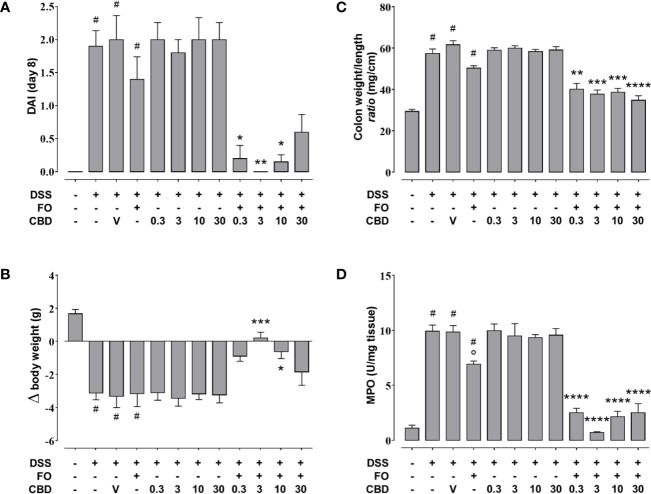
Effect of fish oil (75 mg/mouse) and CBD (0.3–30 mg/kg), both alone and in combination, on DSS-induced inflammation (Experiment 4). Effect of sesame oil (V, 90 µl/mouse, by oral gavage, used as a control), fish oil (FO, 75 mg/mouse corresponding to 70 µl/mouse, by oral gavage), CBD (0.3–30 mg/kg, by oral gavage), and FO (75 mg/mouse) + CBD on disease activity index (DAI) score **(A)**, body weight **(B)**, colon weight/colon length ratio **(C)**, and MPO activity **(D)** in DSS-treated mice (weighting 28–30 g). On the *x*-axis the doses shown are for CBD. Bars are mean ± SEM of 10 animals **(A–C)** and of five tissues **(D)** for each experimental group. Data in **(A)** [F_(11,108)_ = 13.48; p < 0.0001], **(B)** [F_(11,108)_ = 11.13; p < 0.0001], **(C)** [F_(11,108)_ = 44.81; p < 0.0001], and **(D)** [F_(11,48)_ = 48.28; p < 0.0001] were statistically analyzed using one-way ANOVA followed by the Tukey-Kramer multiple comparisons test ^#^p < 0.01–0.0001 vs. control; °p < 0.05 vs. DSS alone; *p < 0.05, **p < 0.01, ***p < 0.001 and ****p < 0.0001 vs. DSS + FO.

FO administration at 75 mg/mouse, numerically reduced colon weight/length ratio and DAI score (**Figures 7A, B**), but this did not reach significant levels, and had no effect on the loss of body weight (**Figure 7C**) in DSS-treated mice. However, it significantly attenuated MPO activity (**Figure 7D**) as observed above. CBD, given by oral gavage at the dose range of 0.3–30 mg/kg did not affect DSS-induced intestinal inflammation, but when administered in FO-treated mice strongly, but variedly, reduced the DAI score (0.3, 3, and 10 mg/mouse only), the colon weight/colon length *ratio* (all doses of CBD tested) the loss of body weight (3 and 10 mg/mouse only) and MPO activity (all doses of CBD tested) (**Figures 7A–D**).

## Discussion

Inflammatory bowel diseases represent widespread and increasing chronic pathological conditions that still lack effective treatments. A plethora of recent studies have highlighted the role of gut dysbiosis in these conditions (Pittayanon et al., 2020). FO and its n-3-PUFAs have been suggested as a potential adjunctive treatment for IBDs, although the clinical data that have been obtained so far come from small cohorts of patients using variable modes of consumption (types of foods or types of formulation) and are therefore still controversial (Marton et al., 2019). On the other hand, while various *Cannabis* preparations have been tested in IBD patients as a potential treatment, purified cannabinoids have been mostly tested in animal models of colitis (Couch et al., 2018). Approximately 15% of IBD patients use *Cannabis* to ameliorate CD symptoms (i.e., abdominal pain, abdominal cramps, joint pain, and diarrhea), although so far there have been only three small placebo-controlled studies regarding the use of *Cannabis* in this disorder, involving 93 subjects altogether. Two of these studies showed overall significant clinical improvement but no amelioration in markers of inflammation (Naftali, 2020). With regard to UC, Kafil et al. recently reviewed the literature of the small clinical trials performed for this type of IBD, and stated that “no firm conclusions regarding the efficacy and safety of *Cannabis* or cannabidiol in adults with active ulcerative colitis can be drawn” (Kafil et al., 2018). This may be due, among other reasons, to the use of small numbers of patients, difficulties in obtaining a real placebo, or the relatively high dropout of patients in the active arm of the study. Nevertheless, several studies in animal models of colitis have highlighted that purified plant-derived cannabinoids can be efficacious, although, of these compounds, purified plant-derived CBD, appeared to be the most studied and the least promising (Borrelli et al., 2009; Borrelli et al., 2013; Romano et al., 2013; Pagano et al., 2016; Couch et al., 2018; Pagano et al., 2019). However, a formulation of highly purified plant-derived CBD was recently approved by the Food and Drug Administration (as Epidiolex^®^) and European Medicines Authority (as Epidyloex^®^, as an adjunctive therapy with clobazam) for the treatment of seizures associated with Dravet and Lennox Gastaut syndromes (two rare forms of pediatric epilepsy); while generally well tolerated, diarrhea is a common adverse event (Pauli et al., 2020). Importantly, while FO and n-3 PUFAs have been tested in experimental colitis also with regard to their effects on the gut microbiota, no preclinical or clinical study has ever been conducted with CBD in this context.

The major finding of the present study is that, when co-administered with inactive or minimally active doses of FO in the DSS murine model of colitis, oral purified botanically derived CBD (≥98%) attenuates inflammation even at relatively low doses. The second major finding of our study is that the potentiating effect on colon inflammation of *per se* ineffective doses of FO and CBD does not appear to be dependent on their effects on the gut microbiota. Finally, we have shown that DSS-induced behavioral alterations, which have been previously described in mice (Reichmann et al., 2015), were not affected by the combination of *per se* ineffective doses of FO and CBD despite its anti-inflammatory effects. However, it must be emphasized that the two behavioral tests used here, although used in previous studies on DSS-induced colitis in mice, cannot be considered sufficient alone to fully evaluate cognitive impairment and anxiety behavior in rodents (Kafil et al., 2018).

When tested in two different sets of experiments of DSS-treated mice, oral FO showed moderate and statistically significant efficacy at counteracting several parameters of DSS-induced colon inflammation, with a maximal effect being observed between 50 and 75 mg/mouse. Conversely, in two separate sets of experiments, oral CBD (0.3–30 mg/kg) produced no significant effect on the same parameters, in partial agreement with previous data obtained using the DNBS and croton oil models of lower and upper intestinal inflammation, where this compound showed only weak activity (Borrelli et al., 2009; Pagano et al., 2016). However, when CBD was administered to mice concurrently treated with FO, it produced an amelioration in most macroscopic measures of inflammation. CBD in conjunction with a *per se* ineffective (20 mg/mouse) dose of FO resulted in partial remission, whereas in conjunction with an effective dose (75 mg/mouse) of FO resulted in full remission. Interestingly, similar results have also been obtained with the DNBS model of colitis (Pagano et al., in press).

DSS-treated mice co-administered with *per se* ineffective doses of FO (20 mg/mouse) and CBD (1 mg/kg) also exhibited a recovery from their elevations in colonic inflammatory cytokine levels and permeability. Such general amelioration was observed both during the apex of inflammation, at d8, and during the RP, at d14. However, this combined treatment did not ameliorate the anxiety-like behavior and cognitive deficits of DSS-treated mice, which are known to be maximal at d8 (Emge et al., 2016) and were only assessed at this time point.

The gut microbiota has been suggested to be both one of the underlying causes of IBDs, when dysfunctional (gut dysbiosis) (Manichanh et al., 2012; Khan et al., 2019; Alam et al., 2020), and a mediator of the anti-inflammatory and therapeutic effects of several different types of pharmacological, nutraceutical and nutritional interventions that proved beneficial against these disorders when investigated in preclinical models (and in the DSS model, in particular) (Wu M. et al., 2019; Fernandez et al., 2020; Gu et al., 2020). Gut dysbiosis is also known to accompany, and possibly underlie, some behavioral disturbances, such as those that are observed in DSS-treated mice, i.e., anxiety and cognitive deficits (Reichmann et al., 2015; Emge et al., 2016). In the present study, only few gut microbiota phyla, families, genera and species were affected by DSS at day 8, possibly suggesting that commensal bacteria may not play an important role in DSS-associated inflammatory and behavioral disturbances under our experimental conditions. Conversely, several taxa were significantly altered at day 14, thus indicating that gut bacteria may play a role in the late effects of DSS, in terms of either residual/resolved inflammation or potential residual behavioral disturbances (which we did not assess at this time point). Interestingly, the anti-inflammatory effect of the combination of *per se* ineffective doses of FO (20 mg/mouse) or CBD (1 mg/kg) was accompanied by changes in several gut bacterial taxa, some of which have been suggested to play a beneficial role in inflammation (see below). However, we found that also when administered alone, these non-anti-inflammatory doses of FO or CBD often similarly modified the relative abundances of commensal bacterial taxa that were either affected or not by DSS at d8. This indicates that the observed effects of FO or CBD on the gut microflora were independent from their effects on inflammation and *vice versa*. Nevertheless, some of these effects on the gut microbiota, as in the case of those of the FO/CBD combination, may have reinforced the anti-inflammatory actions, whereas others, as in the case of those of FO or CBD administered *per se*, may have opposed them. Likewise, the fact that the effects of FO, CBD and their combination on gut microbiota taxa were in some cases only observed at RP (day 14), when resolution of inflammation was ongoing, is indicative of either inflammation-independent effects, or effects that were synergistic/antagonistic with those of endogenous inflammation resolution factors.

In particular, at the phyla level, the Firmicutes:Bacteroidetes ratio was previously reported to be increased in some models of colonic inflammation and in human IBDs (Manichanh et al., 2012; Santoru et al., 2017). In the present study, this biomarker of gut inflammation, typical also of obesity-induced dysbiosis (Ley et al., 2006), was increased by DSS only at RP. The combination of *per se* ineffective doses of FO and CBD, but also the single treatments that exerted no effect on inflammation, counteracted this increase, suggesting that at least the effects of the combination of the two substances may have been reinforced by, but was not dependent on, their action on the Firmicutes:Bacteroidetes ratio.

Of note, the combination of CBD + FO specifically decreased the levels of a small number of bacterial families (*Marinifilaceae* at d8 and *Desulfovibrionaceae* and *Ruminococcaceae* at d14) and one genus (*Odoribacter* at d8) in DSS-treated mice, all of which have previously been shown by others to be modified either in patients with IBDs or their preclinical models. *Desulfovibrionaceae* has been reported to be increased in abundance in the faeces of IBD patients (Berry and Reinisch, 2013) and *Ruminococcaceae* have been reported to be increased in those with UC, but decreased in patients with CD (Morgan et al., 2012; Alam et al., 2020). Pre-clinical studies in addition to ours suggest that these families may have functional roles in IBDs. Indeed, DSS-induced increases of *Desulfovibrionaceae* in mice were abrogated through treatment with the probiotic *Bifidobacterium breve* (Yang and Yang, 2018). However, *Desulfovibrionaceae* and *Ruminococcaceae* were both increased by gentamicin in DSS-treated mice in association with an improved DAI and inflammatory profile (Zhai Z. et al., 2019). Most interestingly, *Ptpn22*^−/−^ mice, which are resistant to faster recovery in response to cohousing-mediated faecal microbiota transfer, have decreased *Desulfovibrionaceae* levels (Spalinger et al., 2019). These data are counterintuitive in the light of our data presented here and given that this family may be an indicator of colitis disease activity. In contrast, in the study by Zhai Z. et al. (2019), *Marinifilaceae* positively correlated with inflammatory status in the mice, which is in line with our study describing significant decreases in this family at d8 by CBD (1 mg/kg) + FO (20 mg/mouse), concomitant to decreased inflammation. Furthermore, both *Marinifilaceae* and *Ruminococcaceae* were decreased in rats fed an acorn-fed cured ham diet (having high levels of the mono-unsaturated fatty acid oleic acid), in conjunction with significant prevention of DSS-induced colitis symptoms (Fernandez et al., 2020).

The short chain fatty acid-producing genus *Odoribacter* is generally considered to play a beneficial role against inflammation and is reduced in CD and UC (Morgan et al., 2012). However, its levels have been shown to increase in response to DSS and decrease in response to electroacupuncture and moxibustion treatment that improved the DAI (Wei et al., 2019). Furthermore, *Odoribacter* is increased in azoxymethane (AOM) and DSS-induced colitis-associated cancer in mice, and its levels are reduced in response to treatment with a probiotic in conjunction with decreased tumor formation (Song et al., 2018). Here we found that the abundance of this genus was not altered by DSS, and was decreased by the FO + CBD combination, an effect that, depending on the role of these bacteria, could either contribute to inhibition of inflammation or represent an adaptive consequence of the latter.

*A. muciniphila*, a species that plays a beneficial role in inflammation and was previously reported to be increased in murine models of IBDs and to mediate the anti-inflammatory effects of several treatments on these models (Bian et al., 2019; Li et al., 2019; Zhai R. et al., 2019; Zhang et al., 2019), was increased by DSS only at d14, suggesting that this effect might represent a potentially adaptive and protective mechanism intervening the resolution of inflammation. Importantly, this effect was rendered statistically significant also at d8 by a combination of *per se* ineffective doses of FO and CBD, but also by the single treatments. This bacterial species may, therefore, participate in some of the beneficial effects of the FO/CBD combination on DSS-induced inflammation, but does not seem to be sufficient to induce such effects. However, the exact role of *A. muciniphila* in colitis remains to be confirmed, as colonization with this gram-negative species has also been shown to increase intestinal inflammation in both specific-pathogen-free and germ-free Il10^−/−^ mice (Seregin et al., 2017). Interestingly, the effects of the treatments observed here on *A. muciniphila* were also observed at the level of its family (*Akkermansiaceae*) and genus (*Akkermansia*), suggesting that this species is the main, if not only, component of its family in the fecal microbiome of DSS-treated mice, and possibly explaining why we could identify this species even though the sequencing method used normally only allows to detect taxa down to the level of genera.

We could also detect at least another species, *P.goldsteinii*, whose abundance, like with *A. muciniphila*, was increased in DSS-treated mice during the RP, and which is known to play a beneficial action in gut inflammation as well obesity (Chang et al., 2019; Wu T. R. et al., 2019). Accordingly, this increase was observed already at d8 following co-treatment with the anti-inflammatory combination of FO and CBD (but, again, also with the single treatments).

Finally, it should be noted that in the present study we have not measured gut motility changes associated with inflammation, an UC clinical phenomenon (Ohama et al., 2007). However, previous studies have demonstrated that CBD does not affect motility under physiological conditions, but it normalizes intestinal motility when this is perturbed by a pro-inflammatory stimulus (Capasso et al., 2008; Lin et al., 2011), and ameliorated motility changes in a TNBS-induced colitis model (Wei et al., 2020). Although CBD has been shown to reduce acetylcholine- and prostaglandin F2α-induced contractions in the isolated ileum (Capasso et al., 2008), there is no evidence that CBD may slow colonic transit under physiological conditions. This is relevant in the light of the observation that drugs able to slow colonic motility (e.g., narcotic, antidiarrheal, or anticholinergic preparations) are contraindicated in toxic megacolon (Gan and Beck, 2003).

In conclusion, we have shown here that FO in combination with CBD can produce strong intestinal anti-inflammatory effects on DSS-induced colitis in mice, and that both FO and CBD, alone or in combination, can also affect the gut microbiota in these mice in a manner partly independent from their anti-inflammatory actions. Future studies should investigate the possibility of using combinations of low doses of FO and CBD, two clinically used substances with very few undesired side effects, for the treatment of colonic inflammation in IBDs. Understanding the functional/biological relevance of the changes induced by the FO/CBD combination on gut microbiota (both murine and human) also merits further research.

## Data Availability Statement

The raw data supporting the conclusions of this article will be made available by the authors, without undue reservation. The datasets analyzed for this study can be found in the NCBI GenBank under BioProject ID PRJNA662783.

## Ethics Statement

The animal study was reviewed and approved by Institutional Animal Ethics Committee of the University of Naples Federico II. The use of animals conformed to guidelines for the safe use and care of experimental animals in accordance with the Italian D.L. no. 116 of 27 January 1992 and associated guidelines in the European Communities Council (86/609/ECC and 2010/63/UE).

## Author Contributions

CS, AI, VD, and FB conceived and designed the study. EP, TV, CC, AC, and OP performed experiments. CS, EP, SL, and FB analyzed data. VD wrote the manuscript. CS, EP, SL, AI, and FB edited the manuscript. All authors contributed to the article and approved the submitted version.

## Funding

This work was supported by GW Research Ltd, Cambridge, UK, the Canada Research Excellence Chair in the Microbiome-Endocannabinoidome Axis in Metabolic Health (CERC-MEND), which is funded by the Tri-Agency of the Canadian Federal Government (The Canadian Institutes of Health Research (CIHR), the Natural Sciences and Engineering Research Council of Canada (NSERC), and the Social Sciences and Humanities Research Council of Canada (SSHRC), as well as by the Canadian Foundation of Innovation (to VD, grant numbers 37392 and 37858) and the Sentinelle Nord-Apogée program (to Université Laval). TV acknowledges the Joint International Research Unit for Chemical and Biomolecular Research on the Microbiome and its impact on Metabolic Health and Nutrition (UMI-MicroMeNu), between Université Laval and the CNR of Italy, which is supported by the Sentinelle Nord program.

## Conflict of Interest

CS, FB, VD, and AI receive research grants from GW Research Ltd, UK. CS was an employee of GW Research Ltd, UK. The authors declare that this study received funding from GW Research Ltd, UK. The funder had the following involvement with the study: has read, edited, and approved of the manuscript for submission.

The remaining authors declare that the research was conducted in the absence of any commercial or financial relationships that could be construed as a potential conflict of interest.
