# Supplementary material for: Fish Oil, Cannabidiol and the Gut Microbiota: An Investigation in a Murine Model of Colitis
Source: Front Pharmacol. 2020 Oct 8;11:585096. doi: 10.3389/fphar.2020.585096 (PMC7580385; doi:10.3389/fphar.2020.585096)
Supplement: Supplementary file 1 [file DataSheet_1.pdf]

Fish oil, cannabidiol and the gut microbiota: an investigation in a murine model of colitis

Cristoforo Silvestri[1+], Ester Pagano[2+], Sébastien Lacroix[3], Tommaso Venneri[2], Claudia Cristiano[2], Antonio Calignano[2], Olga A. Parisi[2], Angelo A. Izzo[2], Vincenzo Di Marzo[1,3,4,5,6,7 *], Francesca Borrelli[2*]

[1]Centre de recherche de l'Institut universitaire de cardiologie et de pneumologie de Québec (IUCPQ), Québec, Canada; Département de médecine, Faculté de Médecine, Université Laval, Québec, Canada

[2]Department of Pharmacy, School of Medicine and Surgery, University of Naples Federico II, Naples, Italy

[3]Institut sur la nutrition et les aliments fonctionnels (INAF), Québec, Canada;

[4]Institute of Biomolecular Chemistry, National Research Council (CNR) of Italy, Pozzuoli, Italy.

[5]Centre Nutriss, École de nutrition, Faculté des sciences de l'agriculture et de l'alimentation (FSAA), Université Laval, Québec, Canada

[6]Joint International Unit between the National Research Council (CNR) of Italy and Université Laval on Chemical and Biomolecular Research on the Microbiome and its Impact on Metabolic Health and Nutrition (UMI-MicroMeNu), Institute of Biomolecular Chemistry, CNR, Pozzuoli (NA), Italy

[7]Canada Research Excellence Chair on the Microbiome-Endocannabinoidome Axis in Metabolic Health (CERC-MEND), Université Laval, Québec, Canada

*Corresponding authors

[+]These authors equally contributed to this study

Running Title: Fish Oil and CBD effects on colitis

Supplementary Figures

Suppl. Figure 1

Effect of fish oil (20 mg/mouse) and CBD (1 mg/kg), both alone and in combination, on the light/dark box test and novel object recognition test in mice with DSS-induced colitis (Experiment 3). For the light/dark box test, the effect on time in dark side (seconds) (A) and the transitions (B) was assessed. For the novel object recognition test, the effect on number of entries on new object (C) and movements (D) was assessed. Effect of sesame oil (V, 90µl/mouse, by oral gavage, used as CBD vehicle), fish oil (FO, 20 mg/mouse, by oral gavage), CBD (1 mg/kg, by oral gavage) and FO+CBD on disease activity index (DAI) score (A) and body weight (B) in control mice (without DSS treatment) and in animals with colitis (induced by DSS). All animals weighed 28-30 g. DAI score was performed in a blinded fashion on day 8 using the scale shown in Table 1 to ensure that colitis had been induced in these mice. Bars are mean± SEM of 10 animals for each experimental group. Data in Figure 7A [$F_{(9,90)}$=18.69; p<0.0001], 7B [$F_{(9,90)}$=21.9; p<0.0001], 7C [$F_{(9,90)}$=31.47; p<0.0001] and 7D [$F_{(9,90)}$=35.8; p<0.0001] were statistically analyzed using one-way ANOVA followed by the Tukey-Kramer multiple comparisons test #p<0.0001 vs control and °p<0.0001 vs vehicle.

Suppl. Figure 2

Effect of fish oil (20 mg/mouse) and CBD (1 mg/kg), both alone and in combination, both at day 8 and day 14, on the relative abundance of some gut bacterial families in faecal samples collected from DSS-treated mice belonging to the same treatments groups as Figures 4-7 (Experiment 3). Only the families that tended to be altered by DSS (i.e. DSS_veh vs. Veh, P<0.1) during inflammation (day 8) (*Streptococcaceae* and *Saccharimonadaceae*), or during remission (day 14) (*Akkermansiaceae* and *Tannerellaceae*) are shown. Data were analysed by two-way ANOVA followed by TukeyHSD post-hoc tests. #, P<0.05 vs. relevant control of the same day. Numbers indicate P values >0.05, <0.1. For F values, please see Supplementary Table 3.

Suppl. Figure 3

Effect of fish oil (20 mg/mouse) and CBD (1 mg/kg), both *per se* and in combination, both at day 8 and day 14, on the relative abundance of some gut bacterial families in faecal samples collected from DSS-treated mice belonging to the same treatments groups as Figures 4-7 (Experiment 3). Only gut bacterial families that were modified by certain treatments from levels observed in DSS-treated mice (DSS_Veh) at day 8 (top two panels) or day 14 (bottom two panels) are shown. Data were analysed by two-way ANOVA followed by TukeyHSD post-hoc tests. #, P<0.05 vs. relevant control of the same day. Numbers indicate P values >0.05 and $\leq$0.1. For F values, please see Supplementary Table 3.

Suppl. Figure 4

Effect of fish oil (20 mg/mouse) and CBD (1 mg/kg), both *per se* and in combination, both at day 8 and day 14, on the relative abundance of some gut bacterial genera in faecal samples collected from DSS-treated mice belonging to the same treatments groups as Figures 4-7 (Experiment 3). Only gut bacterial genera that were affected by DSS (DSS_Veh vs. Veh, P<0.01) at day 8 and/or 14 are shown. Data were analysed by two-way ANOVA followed by TukeyHSD post-hoc tests. #, P<0.05 vs. relevant control of the same day. Numbers indicate P values >0.05, <0.1. For F values, please see Supplementary Table 2.

<u>Suppl. Figure 5</u>

Effect of fish oil (20 mg/mouse) and CBD (1 mg/kg), both *per se* and in combination, both at day 8 and day 14, on the relative abundance of some gut bacterial genera in faecal samples collected from DSS-treated mice belonging to the same treatments groups as Figures 4-7 (Experiment 3). Only gut bacterial genera that were modified by certain treatments from levels observed in DSS-treated mice (DSS_Veh; P<0.01) at day 8 (top 4 panels) or day 14 (bottom 3 panels) are shown. Data were analysed by two-way ANOVA followed by TukeyHSD post-hoc tests. #, P<0.05 vs. relevant control of the same day. Numbers indicate P values >0.05, <0.1. For F values, please see Supplementary Table 2.

Supplementary Fig. 1

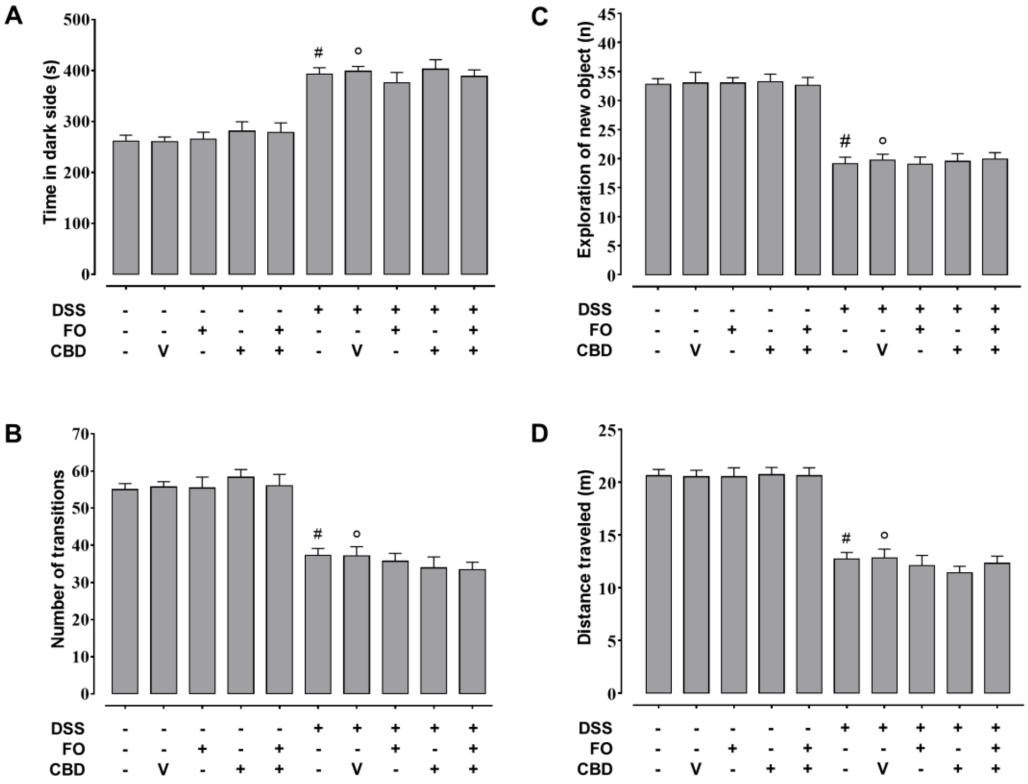

Supplementary Fig. 2

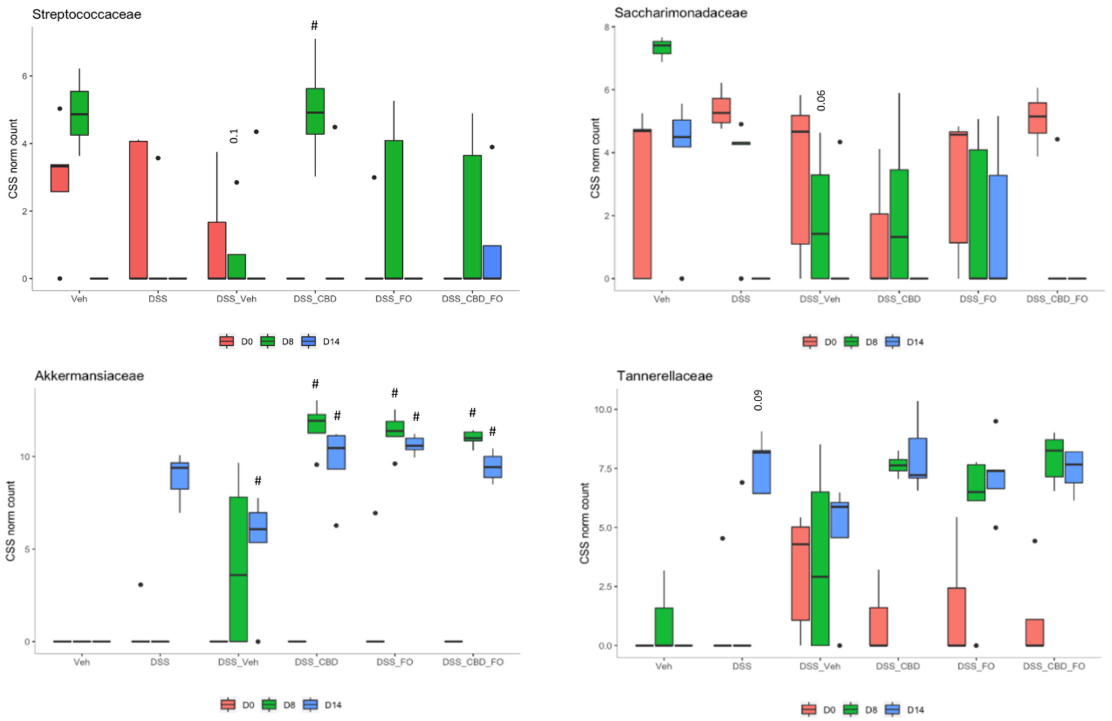

Supplementary Fig. 3

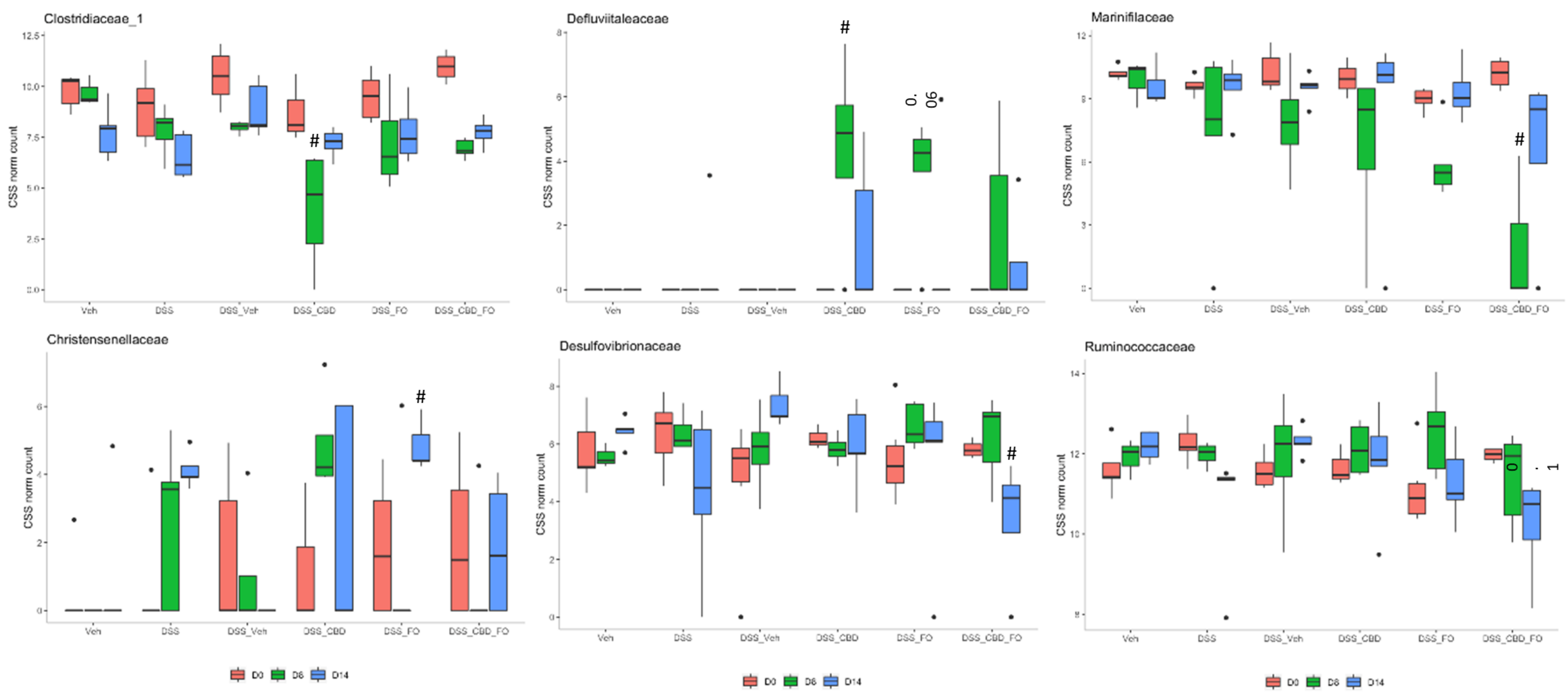

Supplementary Fig. 4

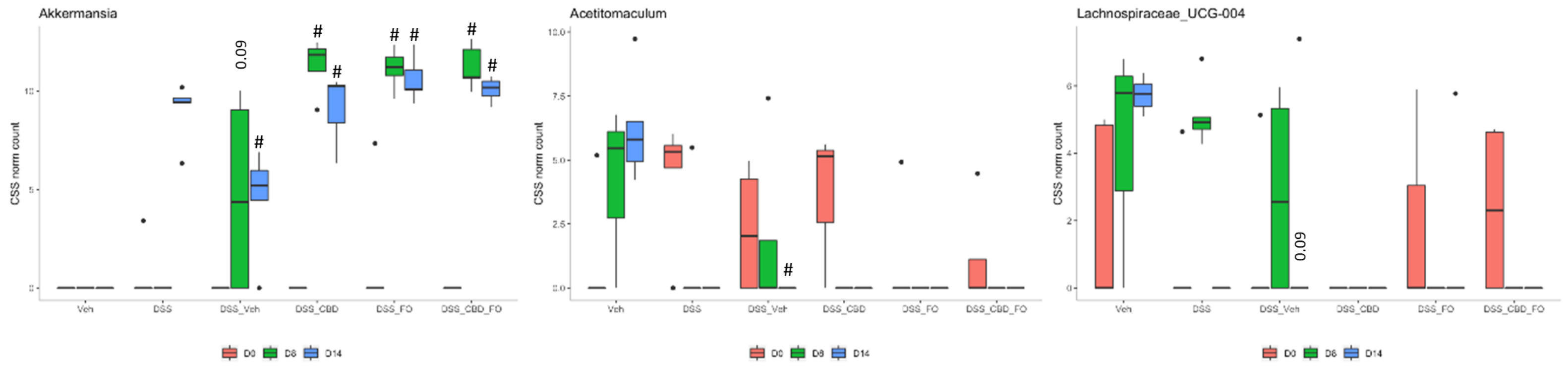

Supplementary Fig. 5

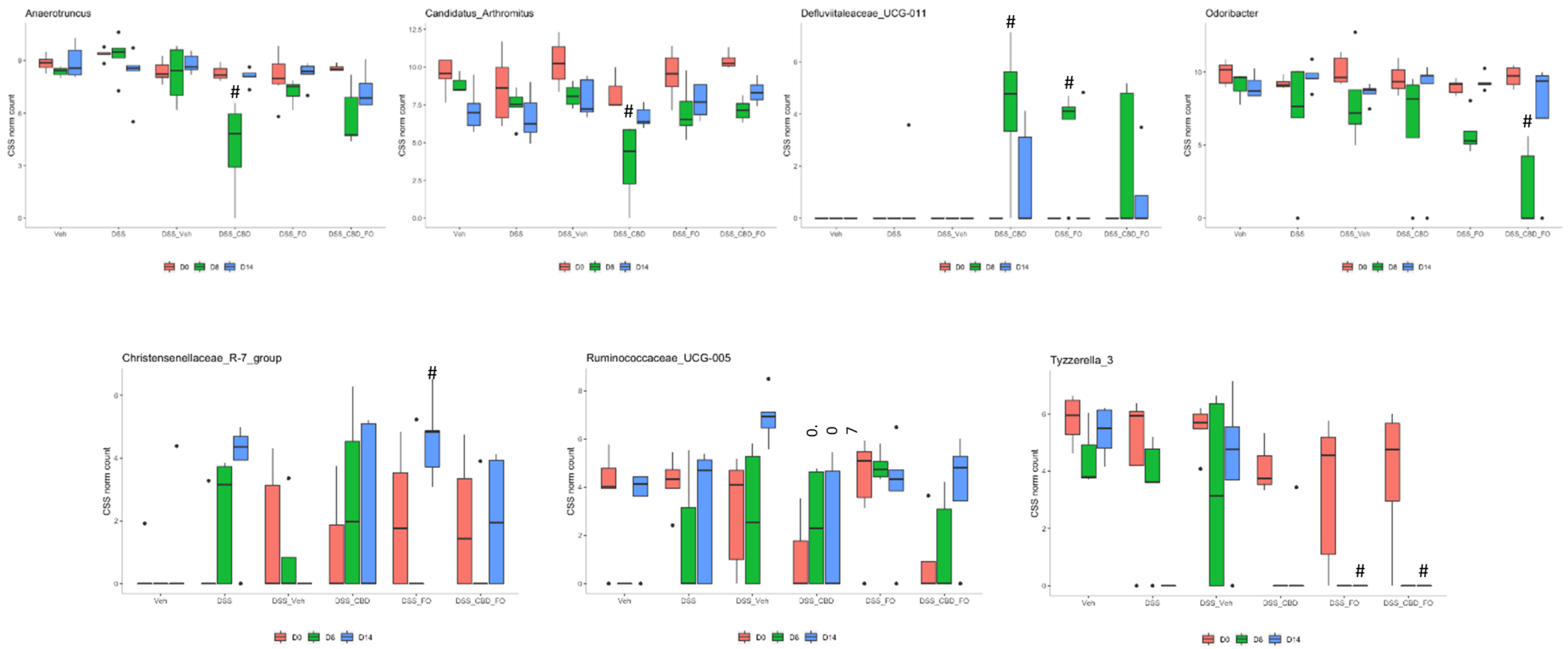

<u>Supplementary Tables</u>

Suppl. Table 1: Statistical details of species affected by treatments

			DSS_Veh vs. Veh			DSS_CBD vs. DSS_Veh			DSS_CBD vs. DSS_Veh			DSS_CBD vs. DSS_Veh		
**Species**	**F Value Group**	**p value Group**	**d0**	**d8**	**d14**	**d0**	**d8**	**d14**	**d0**	**d8**	**d14**	**d0**	**d8**	**d14**
muciniphila	36.27	1.119e-17	ns	ns	0.0004612	ns	9.346e-05	ns	ns	0.0004743	0.01297	ns	0.0006649	ns
goldsteinii	10.91	1.12e-07	ns	ns	0.04791	ns	ns	ns	ns	ns	ns	ns	ns	ns

Suppl. Table 2: Statistical details of genera affected by treatments

			DSS_Veh vs. Veh			DSS_CBD vs. DSS_Veh			DSS_CBD vs. DSS_Veh			DSS_CBD vs. DSS_Veh		
**Genus**	**F Value Group**	**p value Group**	**d0**	**d8**	**d14**	**d0**	**d8**	**d14**	**d0**	**d8**	**d14**	**d0**	**d8**	**d14**
Akkermansia	41.19	5.128e-19	ns	0.09548	0.02074	ns	0.000318	0.01455	ns	0.000185	0.000184	ns	0.000146	0.002601
Acetitomaculum	6.072	0.00011	ns	ns	0.000304	ns	ns	ns	ns	ns	ns	ns	ns	ns
Lachnospiraceae_UCG-004	6.152	9.759e-05	ns	ns	0.09047	ns	ns	ns	ns	ns	ns	ns	ns	ns
Ruminococcus_1	1.087	ns	ns	ns	ns	0.0242	ns	ns	ns	ns	ns	ns	ns	ns
Anaerotruncus	7.608	1.079e-05	ns	ns	ns	ns	0.000532	ns	ns	ns	ns	ns	ns	ns
Candidatus_Arthromitus	6.271	8.106e-05	ns	ns	ns	ns	0.006139	ns	ns	ns	ns	ns	ns	ns
Defluviitaleaceae_UCG-011	4.736	0.000931	ns	ns	ns	ns	0.007143	ns	ns	0.04884	ns	ns	ns	ns
Ruminococcaceae_UCG-005	3.148	0.0131	ns	ns	ns	ns	ns	0.06618	ns	ns	ns	ns	ns	ns
Tyzzerella_3	14.94	8.637e-10	ns	ns	ns	ns	ns	ns	ns	ns	0.02499	ns	ns	0.0472
Christensenellaceae_R-7_group	2.325	0.05248	ns	ns	ns	ns	ns	ns	ns	ns	0.04501	ns	ns	ns
Odoribacter	3.526	0.006935	ns	ns	ns	ns	ns	ns	ns	ns	ns	ns	0.02916	ns

Suppl. Table 3: Statistical details of families affected by treatments

			DSS_Veh vs. Veh			DSS_CBD vs. DSS_Veh			DSS_CBD vs. DSS_Veh			DSS_CBD vs. DSS_Veh		
**Family**	**F Value Group**	**p value Group**	**d0**	**d8**	**d14**	**d0**	**d8**	**d14**	**d0**	**d8**	**d14**	**d0**	**d8**	**d14**
Saccharimonadaceae	3.897	0.003723	ns	0.05972	ns	ns	ns	ns	ns	ns	ns	ns	ns	ns
Streptococcaceae	2.278	0.05676	ns	0.09645	ns	ns	0.03859	ns	ns	ns	ns	ns	ns	ns
Akkermansiaceae	45.39	4.544e-20	ns	ns	0.001293	ns	1.101e-05	0.01417	ns	8.642e-06	0.000765	ns	2.566e-05	0.04992
Tannerellaceae	12.58	1.378e-08	ns	ns	0.09159	ns	ns	ns	ns	ns	ns	ns	ns	ns
Defluviitaleaceae	4.679	0.001023	ns	ns	ns	ns	0.01047	ns	ns	0.06072	ns	ns	ns	ns
Clostridiaceae_1	6.618	4.761e-05	ns	ns	ns	ns	0.01093	ns	ns	ns	ns	ns	ns	ns
no_match	2.68	0.0289	ns	ns	ns	ns	0.01538	ns	ns	0.06117	ns	ns	ns	ns
Christensenellaceae	3.283	0.01043	ns	ns	ns	ns	ns	ns	ns	ns	0.02877	ns	ns	ns
Marinifilaceae	4.487	0.0014	ns	ns	ns	ns	ns	ns	ns	ns	ns	ns	0.0377	ns
Desulfovibrionaceae	0.3941	ns	ns	ns	ns	ns	ns	ns	ns	ns	ns	ns	ns	0.0417
